# 4-Chloro-*N*-(2-pyrid­yl)aniline

**DOI:** 10.1107/S1600536808038658

**Published:** 2008-11-26

**Authors:** Wan Ainna Mardhiah Wan Saffiee, Azila Idris, Zaharah Aiyub, Zanariah Abdullah, Seik Weng Ng

**Affiliations:** a Department of Chemistry, University of Malaya, 50603 Kuala Lumpur, Malaysia

## Abstract

There are two mol­ecules in the asymmetric unit of the title compound, C_11_H_9_ClN_2_, with dihedral angles of 41.84 (12) and 49.24 (12)° between the aromatic ring planes. The two mol­ecules form a dimer *via* a pair of N—H⋯N hydrogen bonds.

## Related literature

For the structures of the two modifications of *N*-(pyrazin-2-yl)aniline, see: Abdullah & Ng (2008 ▶); Wan Saffiee *et al.* (2008 ▶).
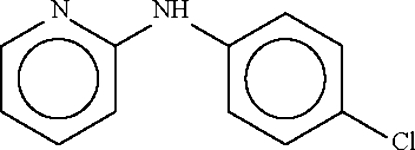


## Experimental

### 

#### Crystal data

C_11_H_9_ClN_2_
*M* *_r_* = 204.65
Monoclinic, 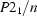

*a* = 15.5096 (4) Å
*b* = 7.5519 (2) Å
*c* = 17.6846 (4) Å
β = 106.284 (2)°
*V* = 1988.25 (9) Å^3^
*Z* = 8
Mo *K*α radiation
μ = 0.34 mm^−1^
*T* = 296 (2) K
0.42 × 0.06 × 0.03 mm

#### Data collection

Bruker SMART APEX CCD diffractometer
Absorption correction: multi-scan (*SADABS*; Sheldrick, 1996 ▶) *T*_min_ = 0.870, *T*_max_ = 0.990
18472 measured reflections
4565 independent reflections
2369 reflections with *I* > 2σ(*I*)
*R*_int_ = 0.041

#### Refinement

*R*[*F* ^2^ > 2σ(*F* ^2^)] = 0.044
*wR*(*F* ^2^) = 0.137
*S* = 1.00
4565 reflections
261 parameters
2 restraints
H atoms treated by a mixture of independent and constrained refinement
Δρ_max_ = 0.15 e Å^−3^
Δρ_min_ = −0.24 e Å^−3^

### 

Data collection: *APEX2* (Bruker, 2007 ▶); cell refinement: *SAINT* (Bruker, 2007 ▶); data reduction: *SAINT*; program(s) used to solve structure: *SHELXS97* (Sheldrick, 2008 ▶); program(s) used to refine structure: *SHELXL97* (Sheldrick, 2008 ▶); molecular graphics: *X-SEED* (Barbour, 2001 ▶); software used to prepare material for publication: *publCIF* (Westrip, 2008 ▶).

## Supplementary Material

Crystal structure: contains datablocks global, I. DOI: 10.1107/S1600536808038658/hb2855sup1.cif

Structure factors: contains datablocks I. DOI: 10.1107/S1600536808038658/hb2855Isup2.hkl

Additional supplementary materials:  crystallographic information; 3D view; checkCIF report

## Figures and Tables

**Table 1:** Hydrogen-bond geometry (Å, °)

*D*—H⋯*A*	*D*—H	H⋯*A*	*D*⋯*A*	*D*—H⋯*A*
N1—H1⋯N4	0.87 (1)	2.16 (1)	3.018 (3)	170 (2)
N3—H3⋯N2	0.86 (1)	2.22 (1)	3.071 (3)	171 (2)

**Fig. 1.**
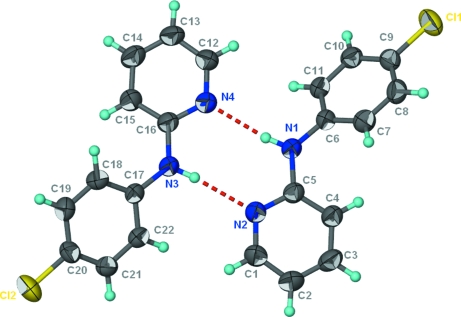
The molecular structure of (I) drawn at the 70% probability level; hydrogen atoms are drawn as spheres of arbitrary radius.

## supplementary crystallographic information

### Comment

For related structures, see: Abdullah & Ng (2008); Wan Saffiee *et al.* (2008).  For the molecular structure of the title compound, (I), see Fig. 1.  For hydrogen bond data, see Table 1.

### Experimental

2-Chloropyrazine (0.11 g, 0.1 mmol) and 4-chloroaniline (0.13 g, 0.1 mmol) were heated at 423–433 K for 5 h. The mixture was cooled and dissolved in water. The solution was extracted with ether. The ether extract was dried over sodium sulfate and the solvent evaporated to yield a dark brown compound. Colourless rods of (I) were separated manually.

### Refinement

The carbon-bound H-atoms were placed in calculated positions (C—H = 0.95 Å) and refined as riding with *U*(H) = 1.2*U*(C). The amino H-atoms were located in a difference map, and were refined with a distance restraint of N–H = 0.88±0.01 Å.

### Crystal data

**Table:**

C_11_H_9_ClN_2_	*F*_000_ = 848
*M**_r_* = 204.65	*D*_x_ = 1.367 Mg m^−^^3^
Monoclinic, *P*2_1_/*n*	Mo *K*α radiation λ = 0.71073 Å
Hall symbol: -P 2yn	Cell parameters from 2114 reflections
*a* = 15.5096 (4) Å	θ = 2.4–21.4º
*b* = 7.5519 (2) Å	µ = 0.34 mm^−^^1^
*c* = 17.6846 (4) Å	*T* = 296 (2) K
β = 106.284 (2)º	Rod, colourless
*V* = 1988.25 (9) Å^3^	0.42 × 0.06 × 0.03 mm
*Z* = 8	

### Data collection

**Table:**

Bruker SMART APEX CCD diffractometer	4565 independent reflections
Radiation source: fine-focus sealed tube	2369 reflections with *I* > 2σ(*I*)
Monochromator: graphite	*R*_int_ = 0.041
*T* = 100(2) K	θ_max_ = 27.5º
ω scans	θ_min_ = 1.6º
Absorption correction: Multi-scan(SADABS; Sheldrick, 1996)	*h* = −20→20
*T*_min_ = 0.870, *T*_max_ = 0.990	*k* = −9→9
18472 measured reflections	*l* = −22→22

### Refinement

**Table:**

Refinement on *F*^2^	Secondary atom site location: difference Fourier map
Least-squares matrix: full	Hydrogen site location: inferred from neighbouring sites
*R*[*F*^2^ > 2σ(*F*^2^)] = 0.045	H atoms treated by a mixture of independent and constrained refinement
*wR*(*F*^2^) = 0.137	*w* = 1/[σ^2^(*F*_o_^2^) + (0.0544*P*)^2^ + 0.3184*P*] where *P* = (*F*_o_^2^ + 2*F*_c_^2^)/3
*S* = 1.00	(Δ/σ)_max_ = 0.001
4565 reflections	Δρ_max_ = 0.15 e Å^−^^3^
261 parameters	Δρ_min_ = −0.24 e Å^−^^3^
2 restraints	Extinction correction: none
Primary atom site location: structure-invariant direct methods	

### Fractional atomic coordinates and isotropic or equivalent isotropic displacement parameters (Å^2^)

**Table:**

	*x*	*y*	*z*	*U*_iso_*/*U*_eq_	
Cl1	0.67109 (6)	0.86166 (11)	0.13818 (4)	0.0915 (3)	
Cl2	0.52605 (6)	0.65436 (13)	0.94056 (4)	0.1027 (3)	
N1	0.67429 (13)	0.7765 (3)	0.47124 (11)	0.0625 (5)	
H1	0.6219 (9)	0.782 (3)	0.4794 (14)	0.074 (8)*	
N2	0.72018 (12)	0.7057 (3)	0.60159 (11)	0.0625 (5)	
N3	0.52408 (13)	0.6934 (3)	0.60693 (11)	0.0651 (6)	
H3	0.5772 (9)	0.691 (3)	0.6004 (13)	0.064 (7)*	
N4	0.48220 (12)	0.7816 (3)	0.47849 (11)	0.0634 (5)	
C1	0.78370 (17)	0.6465 (4)	0.66437 (14)	0.0710 (7)	
H1A	0.7688	0.6375	0.7116	0.085*	
C2	0.86856 (17)	0.5984 (4)	0.66472 (15)	0.0743 (8)	
H2	0.9099	0.5571	0.7102	0.089*	
C3	0.89013 (16)	0.6137 (3)	0.59455 (15)	0.0693 (7)	
H3A	0.9473	0.5828	0.5921	0.083*	
C4	0.82822 (15)	0.6738 (3)	0.52878 (14)	0.0614 (6)	
H4	0.8425	0.6842	0.4813	0.074*	
C5	0.74264 (14)	0.7197 (3)	0.53411 (13)	0.0528 (6)	
C6	0.67699 (14)	0.7960 (3)	0.39292 (12)	0.0517 (5)	
C7	0.74402 (15)	0.8895 (3)	0.37290 (14)	0.0626 (6)	
H7	0.7902	0.9407	0.4122	0.075*	
C8	0.74268 (16)	0.9074 (3)	0.29468 (15)	0.0648 (7)	
H8	0.7889	0.9670	0.2815	0.078*	
C9	0.67292 (17)	0.8368 (3)	0.23664 (14)	0.0590 (6)	
C10	0.60499 (15)	0.7467 (3)	0.25551 (13)	0.0571 (6)	
H10	0.5576	0.7003	0.2160	0.069*	
C11	0.60754 (14)	0.7255 (3)	0.33346 (13)	0.0532 (6)	
H11	0.5619	0.6630	0.3463	0.064*	
C12	0.41850 (17)	0.8195 (4)	0.41237 (14)	0.0756 (8)	
H12	0.4368	0.8569	0.3692	0.091*	
C13	0.32900 (18)	0.8074 (4)	0.40351 (15)	0.0787 (8)	
H13	0.2873	0.8389	0.3565	0.094*	
C14	0.30224 (16)	0.7468 (4)	0.46686 (15)	0.0747 (8)	
H14	0.2415	0.7335	0.4628	0.090*	
C15	0.36502 (15)	0.7066 (3)	0.53537 (14)	0.0654 (7)	
H15	0.3478	0.6647	0.5784	0.078*	
C16	0.45526 (14)	0.7290 (3)	0.54015 (13)	0.0546 (6)	
C17	0.52001 (13)	0.6854 (3)	0.68456 (12)	0.0504 (5)	
C18	0.45807 (15)	0.7767 (3)	0.71257 (13)	0.0573 (6)	
H18	0.4151	0.8470	0.6784	0.069*	
C19	0.45919 (16)	0.7647 (3)	0.79053 (14)	0.0614 (6)	
H19	0.4161	0.8238	0.8084	0.074*	
C20	0.52424 (16)	0.6652 (3)	0.84200 (13)	0.0601 (6)	
C21	0.58698 (15)	0.5743 (3)	0.81581 (13)	0.0592 (6)	
H21	0.6308	0.5071	0.8507	0.071*	
C22	0.58459 (14)	0.5833 (3)	0.73756 (13)	0.0559 (6)	
H22	0.6266	0.5205	0.7197	0.067*	

### Atomic displacement parameters (Å^2^)

**Table:**

	*U*^11^	*U*^22^	*U*^33^	*U*^12^	*U*^13^	*U*^23^
Cl1	0.1128 (6)	0.1099 (6)	0.0642 (4)	0.0274 (5)	0.0455 (4)	0.0168 (4)
Cl2	0.1127 (6)	0.1427 (8)	0.0617 (4)	0.0110 (5)	0.0395 (4)	0.0276 (4)
N1	0.0442 (11)	0.0929 (16)	0.0500 (11)	0.0058 (11)	0.0123 (10)	0.0067 (10)
N2	0.0496 (11)	0.0902 (15)	0.0470 (11)	−0.0002 (10)	0.0125 (9)	−0.0015 (10)
N3	0.0437 (11)	0.1038 (17)	0.0470 (11)	0.0070 (11)	0.0112 (9)	0.0061 (10)
N4	0.0528 (11)	0.0867 (15)	0.0499 (12)	0.0078 (10)	0.0132 (10)	0.0019 (10)
C1	0.0581 (15)	0.104 (2)	0.0467 (14)	−0.0025 (15)	0.0082 (12)	0.0000 (13)
C2	0.0530 (15)	0.101 (2)	0.0594 (16)	0.0018 (14)	0.0003 (12)	0.0038 (14)
C3	0.0453 (13)	0.0847 (19)	0.0717 (17)	0.0065 (13)	0.0062 (13)	−0.0064 (14)
C4	0.0501 (13)	0.0802 (18)	0.0535 (14)	0.0011 (12)	0.0136 (11)	−0.0050 (12)
C5	0.0437 (12)	0.0622 (14)	0.0499 (13)	−0.0034 (11)	0.0086 (11)	−0.0051 (11)
C6	0.0444 (12)	0.0614 (14)	0.0502 (13)	0.0000 (11)	0.0146 (10)	0.0030 (11)
C7	0.0509 (14)	0.0699 (16)	0.0649 (15)	−0.0099 (12)	0.0126 (12)	0.0014 (12)
C8	0.0565 (14)	0.0664 (16)	0.0782 (17)	−0.0024 (12)	0.0301 (13)	0.0124 (13)
C9	0.0627 (15)	0.0628 (16)	0.0556 (14)	0.0137 (12)	0.0231 (12)	0.0072 (11)
C10	0.0489 (13)	0.0655 (16)	0.0547 (14)	0.0020 (11)	0.0107 (11)	−0.0056 (11)
C11	0.0438 (12)	0.0596 (14)	0.0575 (14)	−0.0017 (11)	0.0162 (11)	0.0015 (11)
C12	0.0645 (17)	0.111 (2)	0.0505 (15)	0.0178 (15)	0.0148 (13)	0.0093 (14)
C13	0.0578 (16)	0.119 (2)	0.0516 (15)	0.0208 (16)	0.0031 (13)	0.0020 (15)
C14	0.0464 (14)	0.102 (2)	0.0685 (17)	0.0002 (14)	0.0049 (13)	−0.0065 (15)
C15	0.0488 (13)	0.0883 (18)	0.0560 (14)	−0.0067 (13)	0.0097 (11)	0.0019 (13)
C16	0.0478 (13)	0.0647 (15)	0.0496 (13)	0.0051 (11)	0.0106 (11)	−0.0018 (11)
C17	0.0384 (11)	0.0631 (14)	0.0489 (13)	−0.0045 (10)	0.0108 (10)	0.0009 (10)
C18	0.0499 (13)	0.0631 (15)	0.0555 (14)	0.0051 (11)	0.0094 (11)	0.0066 (11)
C19	0.0562 (14)	0.0704 (17)	0.0622 (15)	0.0023 (12)	0.0244 (12)	0.0011 (12)
C20	0.0603 (15)	0.0692 (16)	0.0526 (14)	−0.0064 (13)	0.0186 (12)	0.0107 (12)
C21	0.0486 (13)	0.0677 (16)	0.0586 (14)	−0.0023 (12)	0.0104 (11)	0.0153 (12)
C22	0.0420 (12)	0.0654 (15)	0.0585 (14)	0.0008 (11)	0.0114 (11)	0.0029 (11)

### Geometric parameters (Å, °)

**Table:**

Cl1—C9	1.744 (2)	C8—C9	1.373 (3)
Cl2—C20	1.737 (2)	C8—H8	0.9300
N1—C5	1.372 (3)	C9—C10	1.371 (3)
N1—C6	1.405 (3)	C10—C11	1.377 (3)
N1—H1	0.865 (10)	C10—H10	0.9300
N2—C1	1.338 (3)	C11—H11	0.9300
N2—C5	1.338 (3)	C12—C13	1.356 (3)
N3—C16	1.378 (3)	C12—H12	0.9300
N3—C17	1.393 (3)	C13—C14	1.378 (3)
N3—H3	0.863 (9)	C13—H13	0.9300
N4—C12	1.333 (3)	C14—C15	1.359 (3)
N4—C16	1.333 (3)	C14—H14	0.9300
C1—C2	1.364 (3)	C15—C16	1.389 (3)
C1—H1A	0.9300	C15—H15	0.9300
C2—C3	1.378 (3)	C17—C18	1.382 (3)
C2—H2	0.9300	C17—C22	1.396 (3)
C3—C4	1.362 (3)	C18—C19	1.377 (3)
C3—H3A	0.9300	C18—H18	0.9300
C4—C5	1.400 (3)	C19—C20	1.377 (3)
C4—H4	0.9300	C19—H19	0.9300
C6—C7	1.382 (3)	C20—C21	1.373 (3)
C6—C11	1.383 (3)	C21—C22	1.376 (3)
C7—C8	1.384 (3)	C21—H21	0.9300
C7—H7	0.9300	C22—H22	0.9300
			
C5—N1—C6	127.0 (2)	C11—C10—H10	120.2
C5—N1—H1	115.4 (16)	C10—C11—C6	120.9 (2)
C6—N1—H1	116.6 (16)	C10—C11—H11	119.5
C1—N2—C5	116.8 (2)	C6—C11—H11	119.5
C16—N3—C17	127.9 (2)	N4—C12—C13	124.7 (2)
C16—N3—H3	115.4 (15)	N4—C12—H12	117.6
C17—N3—H3	116.0 (15)	C13—C12—H12	117.6
C12—N4—C16	117.2 (2)	C12—C13—C14	117.4 (2)
N2—C1—C2	125.3 (2)	C12—C13—H13	121.3
N2—C1—H1A	117.3	C14—C13—H13	121.3
C2—C1—H1A	117.3	C15—C14—C13	119.7 (2)
C1—C2—C3	116.8 (2)	C15—C14—H14	120.2
C1—C2—H2	121.6	C13—C14—H14	120.2
C3—C2—H2	121.6	C14—C15—C16	119.0 (2)
C4—C3—C2	120.4 (2)	C14—C15—H15	120.5
C4—C3—H3A	119.8	C16—C15—H15	120.5
C2—C3—H3A	119.8	N4—C16—N3	114.45 (19)
C3—C4—C5	118.7 (2)	N4—C16—C15	121.9 (2)
C3—C4—H4	120.7	N3—C16—C15	123.6 (2)
C5—C4—H4	120.7	C18—C17—N3	124.0 (2)
N2—C5—N1	114.34 (19)	C18—C17—C22	118.3 (2)
N2—C5—C4	122.0 (2)	N3—C17—C22	117.6 (2)
N1—C5—C4	123.7 (2)	C19—C18—C17	120.7 (2)
C7—C6—C11	118.9 (2)	C19—C18—H18	119.6
C7—C6—N1	122.6 (2)	C17—C18—H18	119.6
C11—C6—N1	118.5 (2)	C18—C19—C20	119.9 (2)
C6—C7—C8	120.3 (2)	C18—C19—H19	120.0
C6—C7—H7	119.9	C20—C19—H19	120.0
C8—C7—H7	119.9	C21—C20—C19	120.5 (2)
C9—C8—C7	119.8 (2)	C21—C20—Cl2	120.07 (18)
C9—C8—H8	120.1	C19—C20—Cl2	119.5 (2)
C7—C8—H8	120.1	C20—C21—C22	119.5 (2)
C10—C9—C8	120.6 (2)	C20—C21—H21	120.2
C10—C9—Cl1	119.87 (19)	C22—C21—H21	120.2
C8—C9—Cl1	119.55 (19)	C21—C22—C17	121.0 (2)
C9—C10—C11	119.5 (2)	C21—C22—H22	119.5
C9—C10—H10	120.2	C17—C22—H22	119.5
			
C5—N2—C1—C2	−0.6 (4)	C16—N4—C12—C13	−0.4 (4)
N2—C1—C2—C3	0.5 (4)	N4—C12—C13—C14	−1.9 (5)
C1—C2—C3—C4	−0.3 (4)	C12—C13—C14—C15	1.8 (4)
C2—C3—C4—C5	0.1 (4)	C13—C14—C15—C16	0.5 (4)
C1—N2—C5—N1	178.4 (2)	C12—N4—C16—N3	−179.4 (2)
C1—N2—C5—C4	0.3 (3)	C12—N4—C16—C15	2.8 (4)
C6—N1—C5—N2	−177.0 (2)	C17—N3—C16—N4	160.2 (2)
C6—N1—C5—C4	0.9 (4)	C17—N3—C16—C15	−22.0 (4)
C3—C4—C5—N2	−0.1 (4)	C14—C15—C16—N4	−2.9 (4)
C3—C4—C5—N1	−177.9 (2)	C14—C15—C16—N3	179.5 (2)
C5—N1—C6—C7	−51.1 (4)	C16—N3—C17—C18	−26.8 (4)
C5—N1—C6—C11	131.9 (3)	C16—N3—C17—C22	155.4 (2)
C11—C6—C7—C8	−1.9 (3)	N3—C17—C18—C19	−178.8 (2)
N1—C6—C7—C8	−178.9 (2)	C22—C17—C18—C19	−1.1 (3)
C6—C7—C8—C9	2.1 (4)	C17—C18—C19—C20	2.0 (4)
C7—C8—C9—C10	−0.8 (4)	C18—C19—C20—C21	−1.5 (4)
C7—C8—C9—Cl1	179.35 (19)	C18—C19—C20—Cl2	178.90 (18)
C8—C9—C10—C11	−0.7 (3)	C19—C20—C21—C22	0.1 (3)
Cl1—C9—C10—C11	179.13 (17)	Cl2—C20—C21—C22	179.70 (18)
C9—C10—C11—C6	0.9 (3)	C20—C21—C22—C17	0.8 (3)
C7—C6—C11—C10	0.4 (3)	C18—C17—C22—C21	−0.4 (3)
N1—C6—C11—C10	177.5 (2)	N3—C17—C22—C21	177.5 (2)

### Hydrogen-bond geometry (Å, °)

**Table:**

*D*—H···*A*	*D*—H	H···*A*	*D*···*A*	*D*—H···*A*
N1—H1···N4	0.87 (1)	2.16 (1)	3.018 (3)	170 (2)
N3—H3···N2	0.86 (1)	2.22 (1)	3.071 (3)	171 (2)

## References

[bb1] Abdullah, Z. & Ng, S. W. (2008). *Acta Cryst.* E**64**, o2106. 10.1107/S1600536808031954 PMC2959673 21580970

[bb2] Barbour, L. J. (2001). *J. Supramol. Chem.* **1**, 189–191.

[bb3] Bruker (2007). *APEX2* and *SAINT* Bruker AXS Inc., Madison, Wisconsin, USA.

[bb4] Sheldrick, G. M. (1996). *SADABS* University of Göttigen, Germany.

[bb5] Sheldrick, G. M. (2008). *Acta Cryst.* A**64**, 112–122. 10.1107/S0108767307043930 18156677

[bb6] Wan Saffiee, W. A. M., Idris, A., Abdullah, Z., Aiyub, Z. & Ng, S. W. (2008). *Acta Cryst.* E**64**, o2105. 10.1107/S1600536808031942 PMC2959679 21580969

[bb7] Westrip, S. P. (2008). *publCIF* In preparation.

